# Supplementary material for: The effect of recent competition between the native Anolis oculatus and the invasive A. cristatellus on display behavior
Source: PeerJ. 2018 Jun 15;6:e4888. doi: 10.7717/peerj.4888 (PMC6005165; doi:10.7717/peerj.4888)
Supplement: Supplemental Information 1 — Table S1. Raw data of the proportion of time spent displaying and the proportion of display-time spent dewlapping for tested male Anolis oculatus and A. cristatellus in allopatry and sympatry (Calibishie, Dominica 2016). All displays were categorized as either dewlap or push-up displays; our metric (“proportion_display_time_ spent_dewlapping“) was the proportion of display time spent in dewlap displays, which is a measure of the relative time spent in the two types of displays. Table S2. Raw data of the canopy openness and the habitat openness for male Anolis oculatus and A. cristatellus in allopatry and sympatry (Calibishie, Dominica 2016), according to the site of sampling. The canopy openness is the number of squares with more than 50% of visible sky, measured with a Ben Meadows spherical densiometer (convex model), from the perch where the lizard was initially observed. The habitat openness is the distance in cm to the closest perch available at the same horizontal plan than where the focal lizard was spotted. [file peerj-06-4888-s001.docx]

Table S1. Raw data of the proportion of time spent displaying and the proportion of display-time spent dewlapping for tested male *Anolis oculatus* and *A. cristatellus* in allopatry and sympatry (Calibishie, Dominica 2016). All displays were categorized as either dewlap or push-up displays; our metric (“proportion_display_time_spent_dewlapping“) was the proportion of display time spent in dewlap displays, which is a measure of the relative time spent in the two types of displays.

| species | context | proportion_time_spent_displaying | proportion_display_time_spent_dewlapping |
| --- | --- | --- | --- |
| A.cristatellus | allopatry | 0.089 | 0.907 |
| A.cristatellus | allopatry | 0.023 | 1.000 |
| A.cristatellus | allopatry | 0.035 | 1.000 |
| A.cristatellus | allopatry | 0.065 | 0.462 |
| A.cristatellus | allopatry | 0.023 | 0.225 |
| A.cristatellus | allopatry | 0.081 | 0.687 |
| A.cristatellus | allopatry | 0.112 | 0.000 |
| A.cristatellus | allopatry | 0.063 | 0.908 |
| A.cristatellus | allopatry | 0.225 | 0.953 |
| A.cristatellus | allopatry | 0.073 | 0.644 |
| A.cristatellus | allopatry | 0.037 | 1.000 |
| A.cristatellus | allopatry | 0.115 | 0.000 |
| A.cristatellus | allopatry | 0.093 | 0.649 |
| A.cristatellus | allopatry | 0.056 | 0.423 |
| A.cristatellus | allopatry | 0.051 | 0.640 |
| A.cristatellus | allopatry | 0.091 | 0.828 |
| A.cristatellus | allopatry | 0.035 | 0.933 |
| A.cristatellus | allopatry | 0.077 | 0.750 |
| A.cristatellus | allopatry | 0.088 | 0.703 |
| A.cristatellus | allopatry | 0.034 | 1.000 |
| A.cristatellus | allopatry | 0.079 | 0.242 |
| A.cristatellus | allopatry | 0.036 | 0.485 |
| A.cristatellus | allopatry | 0.099 | 0.182 |
| A.cristatellus | sympatry | 0.073 | 0.354 |
| A.cristatellus | sympatry | 0.050 | 0.000 |
| A.cristatellus | sympatry | 0.053 | 0.000 |
| A.cristatellus | sympatry | 0.076 | 0.257 |
| A.cristatellus | sympatry | 0.051 | 0.545 |
| A.cristatellus | sympatry | 0.023 | 0.000 |
| A.cristatellus | sympatry | 0.066 | 1.000 |
| A.cristatellus | sympatry | 0.103 | 0.328 |
| A.cristatellus | sympatry | 0.033 | 0.000 |
| A.cristatellus | sympatry | 0.035 | 0.247 |
| A.cristatellus | sympatry | 0.021 | 0.471 |
| A.cristatellus | sympatry | 0.103 | 0.643 |
| A.cristatellus | sympatry | 0.105 | 1.000 |
| A.cristatellus | sympatry | 0.020 | 0.000 |
| A.cristatellus | sympatry | 0.031 | 0.000 |
| A.cristatellus | sympatry | 0.017 | 0.000 |
| A.cristatellus | sympatry | 0.071 | 0.742 |
| A.cristatellus | sympatry | 0.023 | 0.759 |
| A.cristatellus | sympatry | 0.052 | 0.271 |
| A.cristatellus | sympatry | 0.009 | 1.000 |
| A.cristatellus | sympatry | 0.010 | 0.000 |
| A.cristatellus | sympatry | 0.025 | 0.000 |
| A.cristatellus | sympatry | 0.032 | 0.000 |
| A.cristatellus | sympatry | 0.075 | 0.736 |
| A.cristatellus | sympatry | 0.021 | 1.000 |
| A.cristatellus | sympatry | 0.050 | 0.478 |
| A.cristatellus | sympatry | 0.144 | 1.000 |
| A.cristatellus | sympatry | 0.035 | 0.549 |
| A.cristatellus | sympatry | 0.066 | 0.582 |
| A.cristatellus | sympatry | 0.098 | 0.353 |
| A.oculatus | allopatry | 0.113 | 1.000 |
| A.oculatus | allopatry | 0.093 | 0.851 |
| A.oculatus | allopatry | 0.058 | 0.876 |
| A.oculatus | allopatry | 0.131 | 0.995 |
| A.oculatus | allopatry | 0.114 | 1.000 |
| A.oculatus | allopatry | 0.145 | 1.000 |
| A.oculatus | allopatry | 0.627 | 0.642 |
| A.oculatus | allopatry | 0.024 | 1.000 |
| A.oculatus | allopatry | 0.062 | 0.972 |
| A.oculatus | allopatry | 0.132 | 0.260 |
| A.oculatus | allopatry | 0.047 | 1.000 |
| A.oculatus | allopatry | 0.106 | 1.000 |
| A.oculatus | allopatry | 0.021 | 0.956 |
| A.oculatus | allopatry | 0.003 | 1.000 |
| A.oculatus | allopatry | 0.139 | 0.806 |
| A.oculatus | allopatry | 0.041 | 1.000 |
| A.oculatus | allopatry | 0.101 | 0.960 |
| A.oculatus | allopatry | 0.013 | 1.000 |
| A.oculatus | allopatry | 0.104 | 0.956 |
| A.oculatus | allopatry | 0.031 | 0.922 |
| A.oculatus | allopatry | 0.057 | 0.991 |
| A.oculatus | allopatry | 0.017 | 1.000 |
| A.oculatus | allopatry | 0.021 | 0.765 |
| A.oculatus | allopatry | 0.031 | 1.000 |
| A.oculatus | allopatry | 0.133 | 0.954 |
| A.oculatus | allopatry | 0.040 | 0.725 |
| A.oculatus | allopatry | 0.044 | 0.898 |
| A.oculatus | allopatry | 0.019 | 1.000 |
| A.oculatus | allopatry | 0.115 | 0.774 |
| A.oculatus | allopatry | 0.028 | 1.000 |
| A.oculatus | allopatry | 0.125 | 1.000 |
| A.oculatus | sympatry | 0.163 | 0.872 |
| A.oculatus | sympatry | 0.071 | 1.000 |
| A.oculatus | sympatry | 0.113 | 1.000 |
| A.oculatus | sympatry | 0.020 | 1.000 |
| A.oculatus | sympatry | 0.038 | 1.000 |
| A.oculatus | sympatry | 0.130 | 1.000 |
| A.oculatus | sympatry | 0.032 | 1.000 |
| A.oculatus | sympatry | 0.106 | 1.000 |
| A.oculatus | sympatry | 0.190 | 0.987 |
| A.oculatus | sympatry | 0.029 | 0.936 |
| A.oculatus | sympatry | 0.169 | 0.932 |
| A.oculatus | sympatry | 0.108 | 0.825 |
| A.oculatus | sympatry | 0.041 | 1.000 |
| A.oculatus | sympatry | 0.017 | 0.277 |
| A.oculatus | sympatry | 0.070 | 1.000 |
| A.oculatus | sympatry | 0.098 | 0.923 |
| A.oculatus | sympatry | 0.073 | 1.000 |
| A.oculatus | sympatry | 0.029 | 0.805 |
| A.oculatus | sympatry | 0.056 | 0.810 |
| A.oculatus | sympatry | 0.008 | 0.774 |
| A.oculatus | sympatry | 0.079 | 0.835 |
| A.oculatus | sympatry | 0.057 | 0.564 |
| A.oculatus | sympatry | 0.010 | 1.000 |
| A.oculatus | sympatry | 0.074 | 1.000 |
| A.oculatus | sympatry | 0.048 | 0.843 |
| A.oculatus | sympatry | 0.221 | 0.971 |
| A.oculatus | sympatry | 0.510 | 0.916 |
| A.oculatus | sympatry | 0.122 | 0.726 |
| A.oculatus | sympatry | 0.012 | 1.000 |
| A.oculatus | sympatry | 0.032 | 0.516 |
| A.oculatus | sympatry | 0.094 | 0.941 |
| A.oculatus | sympatry | 0.041 | 0.708 |
| A.oculatus | sympatry | 0.023 | 1.000 |
| A.oculatus | sympatry | 0.040 | 1.000 |
| A.oculatus | sympatry | 0.018 | 0.861 |
| A.oculatus | sympatry | 0.010 | 1.000 |
| A.oculatus | sympatry | 0.140 | 0.941 |
| A.oculatus | sympatry | 0.078 | 0.962 |

Table S2. Raw data of the canopy openness and the habitat openness for male *Anolis oculatus* and *A. cristatellus* in allopatry and sympatry (Calibishie, Dominica, 2016), according to the site of sampling. The canopy openness is the number of squares with more than 50% of visible sky, measured with a Ben Meadows spherical densitometer (convex model), from the perch where the lizard was initially observed. The habitat openness is the distance in cm to the closest perch available at the same horizontal plan than where the focal lizard was spotted.

| site | species | context | canopy_openess | habitat_openess |
| --- | --- | --- | --- | --- |
| S2 | A.cristatellus | allopatry | 17 | 190 |
| S2 | A.cristatellus | allopatry | 4 | 10 |
| S2 | A.cristatellus | allopatry | 0 | 20 |
| S2 | A.cristatellus | allopatry | 0 | 15 |
| S2 | A.cristatellus | allopatry | 0 | 2 |
| S2 | A.cristatellus | allopatry | 3 | 45 |
| S2 | A.cristatellus | allopatry | 0 | 32 |
| S2 | A.cristatellus | allopatry | 15 | 62 |
| S2 | A.cristatellus | allopatry | 0 | 20 |
| S2 | A.cristatellus | allopatry | 0 | 37 |
| S2 | A.cristatellus | allopatry | 8 | 48 |
| S2 | A.cristatellus | allopatry | 5 | 30 |
| S2 | A.cristatellus | allopatry | 0 | 17 |
| S7 | A.cristatellus | allopatry | 0 | 10 |
| S7 | A.cristatellus | allopatry | 1 | 60 |
| S7 | A.cristatellus | allopatry | 24 | 7 |
| S7 | A.cristatellus | allopatry | 1 | 15 |
| S7 | A.cristatellus | allopatry | 3 | 24 |
| S7 | A.cristatellus | allopatry | 10 | 45 |
| S7 | A.cristatellus | allopatry | 2 | 110 |
| S7 | A.cristatellus | allopatry | 7 | 20 |
| S7 | A.cristatellus | allopatry | 7 | 33 |
| S7 | A.cristatellus | allopatry | 22 | 21 |
| S7 | A.cristatellus | allopatry | 5 | 9 |
| S7 | A.cristatellus | allopatry | 8 | 25 |
| S7 | A.cristatellus | allopatry | 4 | 20 |
| S7 | A.cristatellus | allopatry | 3 | 13 |
| S7 | A.cristatellus | allopatry | 3 | 10 |
| S7 | A.cristatellus | allopatry | 0 | 15 |
| S7 | A.cristatellus | allopatry | 4 | 29 |
| S7 | A.cristatellus | allopatry | 0 | 50 |
| S3 | A.cristatellus | sympatry | 1 | 15 |
| S3 | A.cristatellus | sympatry | 16 | 14 |
| S3 | A.cristatellus | sympatry | 4 | 20 |
| S3 | A.cristatellus | sympatry | 5 | 23 |
| S3 | A.cristatellus | sympatry | 13 | 7 |
| S3 | A.cristatellus | sympatry | 2 | 29 |
| S3 | A.cristatellus | sympatry | 23 | 30 |
| S3 | A.cristatellus | sympatry | 12 | 54 |
| S3 | A.cristatellus | sympatry | 3 | 180 |
| S3 | A.cristatellus | sympatry | 23 | 128 |
| S3 | A.cristatellus | sympatry | 2 | 18 |
| S3 | A.cristatellus | sympatry | 4 | 50 |
| S5 | A.cristatellus | sympatry | 3 | 70 |
| S5 | A.cristatellus | sympatry | 6 | 50 |
| S5 | A.cristatellus | sympatry | 1 | 38 |
| S5 | A.cristatellus | sympatry | 1 | 10 |
| S5 | A.cristatellus | sympatry | 17 | 20 |
| S5 | A.cristatellus | sympatry | 0 | 30 |
| S5 | A.cristatellus | sympatry | 0 | 34 |
| S5 | A.cristatellus | sympatry | 0 | 13 |
| S5 | A.cristatellus | sympatry | 2 | 18 |
| S5 | A.cristatellus | sympatry | 5 | 11 |
| S5 | A.cristatellus | sympatry | 1 | 34 |
| S5 | A.cristatellus | sympatry | 13 | 65 |
| S5 | A.cristatellus | sympatry | 4 | 20 |
| S5 | A.cristatellus | sympatry | 12 | 52 |
| S6 | A.cristatellus | sympatry | 3 | 44 |
| S6 | A.cristatellus | sympatry | 1 | 21 |
| S6 | A.cristatellus | sympatry | 0 | 85 |
| S6 | A.cristatellus | sympatry | 0 | 9 |
| S6 | A.cristatellus | sympatry | 0 | 54 |
| S6 | A.cristatellus | sympatry | 4 | 56 |
| S6 | A.cristatellus | sympatry | 19 | 143 |
| S6 | A.cristatellus | sympatry | 0 | 35 |
| S6 | A.cristatellus | sympatry | 13 | 35 |
| S6 | A.cristatellus | sympatry | 17 | 26 |
| S6 | A.cristatellus | sympatry | 8 | 110 |
| S6 | A.cristatellus | sympatry | 7 | 20 |
| S6 | A.cristatellus | sympatry | 1 | 83 |
| S6 | A.cristatellus | sympatry | 1 | 20 |
| S6 | A.cristatellus | sympatry | 6 | 40 |
| S6 | A.cristatellus | sympatry | 24 | 12 |
| S6 | A.cristatellus | sympatry | 9 | 83 |
| S6 | A.cristatellus | sympatry | 4 | 25 |
| S6 | A.cristatellus | sympatry | 17 | 40 |
| S6 | A.cristatellus | sympatry | 5 | 5 |
| S6 | A.cristatellus | sympatry | 19 | 120 |
| S9 | A.cristatellus | sympatry | 0 | 10 |
| S9 | A.cristatellus | sympatry | 3 | 97 |
| S9 | A.cristatellus | sympatry | 3 | 20 |
| S1 | A.oculatus | allopatry | 4 | 20 |
| S1 | A.oculatus | allopatry | 17 | 110 |
| S1 | A.oculatus | allopatry | 20 | 49 |
| S1 | A.oculatus | allopatry | 0 | 42 |
| S1 | A.oculatus | allopatry | 10 | 40 |
| S1 | A.oculatus | allopatry | 0 | 24 |
| S1 | A.oculatus | allopatry | 22 | 135 |
| S1 | A.oculatus | allopatry | 16 | 70 |
| S1 | A.oculatus | allopatry | 17 | 4 |
| S1 | A.oculatus | allopatry | 5 | 38 |
| S1 | A.oculatus | allopatry | 6 | 14 |
| S1 | A.oculatus | allopatry | 17 | 117 |
| S1 | A.oculatus | allopatry | 0 | 22 |
| S1 | A.oculatus | allopatry | 4 | 157 |
| S1 | A.oculatus | allopatry | 11 | 5 |
| S1 | A.oculatus | allopatry | 7 | 26 |
| S1 | A.oculatus | allopatry | 10 | 26 |
| S1 | A.oculatus | allopatry | 10 | 17 |
| S1 | A.oculatus | allopatry | 19 | 60 |
| S1 | A.oculatus | allopatry | 20 | 5 |
| S1 | A.oculatus | allopatry | 4 | 110 |
| S1 | A.oculatus | allopatry | 2 | 150 |
| S1 | A.oculatus | allopatry | 14 | 9 |
| S1 | A.oculatus | allopatry | 0 | 12 |
| S1 | A.oculatus | allopatry | 5 | 70 |
| S1 | A.oculatus | allopatry | 15 | 50 |
| S1 | A.oculatus | allopatry | 5 | 7 |
| S1 | A.oculatus | allopatry | 2 | 22 |
| S1 | A.oculatus | allopatry | 0 | 32 |
| S1 | A.oculatus | allopatry | 0 | 105 |
| S1 | A.oculatus | allopatry | 0 | 26 |
| S1 | A.oculatus | allopatry | 1 | 56 |
| S1 | A.oculatus | allopatry | 1 | 50 |
| S8 | A.oculatus | allopatry | 9 | 10 |
| S8 | A.oculatus | allopatry | 2 | 14 |
| S8 | A.oculatus | allopatry | 0 | 100 |
| S8 | A.oculatus | allopatry | 0 | 15 |
| S8 | A.oculatus | allopatry | 11 | 203 |
| S8 | A.oculatus | allopatry | 4 | 150 |
| S8 | A.oculatus | allopatry | 6 | 40 |
| S8 | A.oculatus | allopatry | 0 | 25 |
| S8 | A.oculatus | allopatry | 0 | 100 |
| S8 | A.oculatus | allopatry | 1 | 15 |
| S8 | A.oculatus | allopatry | 1 | 21 |
| S8 | A.oculatus | allopatry | 9 | 21 |
| S8 | A.oculatus | allopatry | 4 | 65 |
| S8 | A.oculatus | allopatry | 0 | 6 |
| S8 | A.oculatus | allopatry | 0 | 17 |
| S8 | A.oculatus | allopatry | 17 | 165 |
| S8 | A.oculatus | allopatry | 3 | 60 |
| S8 | A.oculatus | allopatry | 9 | 80 |
| S8 | A.oculatus | allopatry | 12 | 50 |
| S8 | A.oculatus | allopatry | 1 | 47 |
| S8 | A.oculatus | allopatry | 2 | 23 |
| S8 | A.oculatus | allopatry | 0 | 27 |
| S8 | A.oculatus | allopatry | 0 | 18 |
| S8 | A.oculatus | allopatry | 0 | 55 |
| S3 | A.oculatus | sympatry | 3 | 8 |
| S3 | A.oculatus | sympatry | 8 | 30 |
| S3 | A.oculatus | sympatry | 2 | 45 |
| S3 | A.oculatus | sympatry | 3 | 30 |
| S3 | A.oculatus | sympatry | 0 | 20 |
| S3 | A.oculatus | sympatry | 1 | 41 |
| S3 | A.oculatus | sympatry | 0 | 27 |
| S3 | A.oculatus | sympatry | 6 | 24 |
| S3 | A.oculatus | sympatry | NA | 160 |
| S3 | A.oculatus | sympatry | 10 | 140 |
| S3 | A.oculatus | sympatry | 20 | 196 |
| S3 | A.oculatus | sympatry | 0 | 40 |
| S3 | A.oculatus | sympatry | 0 | 18 |
| S3 | A.oculatus | sympatry | 1 | 50 |
| S4 | A.oculatus | allopatry | 19 | 84 |
| S4 | A.oculatus | allopatry | 7 | 40 |
| S4 | A.oculatus | allopatry | 0 | 13 |
| S4 | A.oculatus | allopatry | 21 | 35 |
| S4 | A.oculatus | allopatry | 19 | 19 |
| S4 | A.oculatus | allopatry | 2 | 16 |
| S4 | A.oculatus | allopatry | 18 | 250 |
| S4 | A.oculatus | allopatry | 7 | 21 |
| S4 | A.oculatus | allopatry | 0 | 49 |
| S4 | A.oculatus | allopatry | 8 | 198 |
| S4 | A.oculatus | allopatry | 3 | 47 |
| S4 | A.oculatus | allopatry | 6 | 227 |
| S4 | A.oculatus | allopatry | 1 | 35 |
| S4 | A.oculatus | allopatry | 19 | 143 |
| S4 | A.oculatus | allopatry | 3 | 29 |
| S4 | A.oculatus | allopatry | 2 | 8 |
| S4 | A.oculatus | allopatry | 4 | 230 |
| S4 | A.oculatus | allopatry | 3 | 240 |
| S5 | A.oculatus | sympatry | 0 | 11 |
| S5 | A.oculatus | sympatry | 15 | 6 |
| S5 | A.oculatus | sympatry | 14 | 170 |
| S5 | A.oculatus | sympatry | 9 | 31 |
| S5 | A.oculatus | sympatry | 5 | 17 |
| S5 | A.oculatus | sympatry | 7 | 17 |
| S5 | A.oculatus | sympatry | 10 | 240 |
| S5 | A.oculatus | sympatry | 8 | 64 |
| S5 | A.oculatus | sympatry | 6 | 123 |
| S5 | A.oculatus | sympatry | 7 | 19 |
| S5 | A.oculatus | sympatry | 4 | 20 |
| S5 | A.oculatus | sympatry | 3 | 29 |
| S5 | A.oculatus | sympatry | 3 | 60 |
| S5 | A.oculatus | sympatry | 1 | 28 |
| S5 | A.oculatus | sympatry | 0 | 19 |
| S5 | A.oculatus | sympatry | 10 | 24 |
| S5 | A.oculatus | sympatry | 0 | 32 |
| S5 | A.oculatus | sympatry | 14 | 30 |
| S5 | A.oculatus | sympatry | 3 | 30 |
| S5 | A.oculatus | sympatry | 4 | 27 |
| S5 | A.oculatus | sympatry | 0 | 45 |
| S6 | A.oculatus | sympatry | 0 | 41 |
| S6 | A.oculatus | sympatry | 0 | 40 |
| S6 | A.oculatus | sympatry | 0 | 36 |
| S6 | A.oculatus | sympatry | 0 | 46 |
| S6 | A.oculatus | sympatry | 0 | 70 |
| S6 | A.oculatus | sympatry | 8 | 30 |
| S6 | A.oculatus | sympatry | 5 | 30 |
| S6 | A.oculatus | sympatry | 2 | 12 |
| S6 | A.oculatus | sympatry | 0 | 21 |
| S6 | A.oculatus | sympatry | 24 | 78 |
| S6 | A.oculatus | sympatry | 9 | 60 |
| S6 | A.oculatus | sympatry | 2 | 60 |
| S6 | A.oculatus | sympatry | 7 | 70 |
| S6 | A.oculatus | sympatry | 5 | 60 |
| S9 | A.oculatus | sympatry | 5 | 41 |
| S9 | A.oculatus | sympatry | 0 | 120 |
| S9 | A.oculatus | sympatry | 0 | 44 |
| S9 | A.oculatus | sympatry | 2 | 35 |
| S9 | A.oculatus | sympatry | 6 | 35 |
| S9 | A.oculatus | sympatry | 0 | 46 |
| S9 | A.oculatus | sympatry | 1 | 20 |
| S9 | A.oculatus | sympatry | 1 | 30 |
| S9 | A.oculatus | sympatry | 18 | 30 |
| S9 | A.oculatus | sympatry | 7 | 36 |
| S9 | A.oculatus | sympatry | 1 | 11 |
| S9 | A.oculatus | sympatry | 9 | 13 |
| S9 | A.oculatus | sympatry | 8 | 20 |
| S9 | A.oculatus | sympatry | 2 | 17 |
| S9 | A.oculatus | sympatry | 7 | 34 |
| S9 | A.oculatus | sympatry | 15 | 220 |
| S9 | A.oculatus | sympatry | 4 | 18 |
| S9 | A.oculatus | sympatry | 6 | 32 |
| S9 | A.oculatus | sympatry | 2 | 24 |
| S9 | A.oculatus | sympatry | 7 | 63 |
| S9 | A.oculatus | sympatry | 5 | 210 |
| S9 | A.oculatus | sympatry | 6 | 30 |
| S9 | A.oculatus | sympatry | 0 | 150 |
